# Burden of drug use disorders from 1990 to 2021 and projection to 2050 in China: findings from the 2021 global burden of disease study

**DOI:** 10.3389/fphar.2025.1625873

**Published:** 2025-09-22

**Authors:** Shuzheng Yu, Yanting Chen, Shaohua Huang, Bing Wang, Qingrong Wu

**Affiliations:** ^1^ Department of Neurosurgery, Ganzhou Hospital-Nanfang Hospital, Southern Medical University, Ganzhou, Jiangxi, China; ^2^ Rheumatology Department, The First Affiliated Hospital of Guangdong Pharmaceutical University, Guangzhou, Guangdong, China; ^3^ Jiangxi University of Chinese Medicine, Nanchang, Jiangxi, China; ^4^ Department of Nephrology, Ganzhou People’s Hospital, Ganzhou, Jiangxi, China; ^5^ Department of Pharmacy, Ganzhou Key Laboratory of Respiratory Diseases, Ganzhou Institute of Respiratory Diseases, The Fifth People’s Hospital of Ganzhou, Ganzhou, Jiangxi, China

**Keywords:** drug use disorders, global burden of disease, disability-adjusted life years, projection, epidemiologic studies

## Abstract

**Background:**

Drug use disorders (DUDs) represent a major public health challenge globally, including China. This study investigated the impact of DUDs in China over the past 3 decades and examined the long-term trends in their epidemiological characteristics.

**Methods:**

Data were obtained from the 2021 Global Burden of Disease (GBD) study. The burden of DUDs was assessed through Disability-Adjusted Life Years (DALYs), Years of Life Lost (YLLs), and Years Lived with Disability (YLDs). Joinpoint analysis was used to compute the Average Annual Percentage Change (AAPC), and Age-Period-Cohort analyses were conducted to illustrate trends in the burden of DUDs. A Bayesian Age-Period-Cohort model was fitted to forecast the anticipated burden.

**Results:**

From 1990 to 2021, the Age-Standardized Incidence Rate (ASIR), Age-Standardized Prevalence Rate (ASPR), and Age-Standardized Mortality Rate (ASMR) for DUDs in China demonstrated a consistent decline, with AAPC values of −0.76% (95% confidence interval [CI]: 0.83% to −0.69%), −1.05% (95% CI: 1.25% to −0.84%), and −4.41% (95% CI: 4.75% to −4.08%), respectively. Analysis of temporal trends indicated that the ASIR and ASPR for DUDs peaked between 1990 and 2000, followed by a variable decline, with a minor uptick noted from 2015 to 2021. The age-standardized DALY rates reached their zenith during 1990–2000. Additionally, analysis by age group indicated that from 1990 to 2021, the ASIR and ASPR for DUDs were highest among individuals aged 20–24 years, followed by a fluctuating decline. Gender-based analysis indicated that throughout this period, disease burden indicators for males consistently surpassed those for females. Projections from the Bayesian Age-Period-Cohort model suggest the ASIR will increase by 3.91% annually from 2022 to 2050, with growth rates of 4.54% for males and 3.25% for females.

**Conclusion:**

Between 1990 and 2021, China experienced a decline in the overall burden of DUDs. However, high incidence and prevalence rates persist, signifying an ongoing significant impact. By 2050, both incidence and prevalence rates of drug abuse are expected to increase significantly, necessitating a focus on the male population and the development of targeted prevention and intervention strategies.

## 1 Introduction

Drug use disorders (DUDs) refer to the misuse of certain substances characterized by dependency or the potential for dependency, pursued for specific psychological effects rather than medical purposes. The core feature of DUDs is substance dependence, manifested through a strong craving for the substance, impaired control over its use, withdrawal symptoms, tolerance, and significant time spent on activities related to the substance. DUDs typically encompass opioid use disorder, cocaine use disorder, amphetamine use disorder, and cannabis use disorder. These disorders can precipitate severe psychological and physiological consequences, as well as substantial social issues (Pan et al., 2020; Lu et al., 2023), which may include cognitive impairment, suicidal tendencies, decreased quality of life, and increased risk of infectious diseases (Guo et al., 2019; Skarstein et al., 2023). According to the 2021 Global Burden of Disease, Injuries, and Risk Factors Study (GBD) data, DUDs constitute a major public health threat, exacerbating the global disease burden and driving a sharp increase in mortality (Zhang et al., 2024; Lei et al., 2025). The 2019 GBD data indicated that DUDs ranked among the top 20 causes of Disability-Adjusted Life Years (DALYs) for individuals aged 10–49 (7). In 2021, the global prevalence of DUDs reached 53,115,936 cases, reflecting a 35.50% increase since 1990, and it is projected to continue rising over the next 25 years (Dongying et al., 2025). The World Drug Report 2023 indicates that over 296 million people worldwide used drugs in 2021, with the number of individuals suffering from DUDs reaching 39.5 million—a 45% increase over the past decade (Crime UNOoDa, 2023). Despite this alarming trend, only one in five individuals with drug-related disorders receives treatment, and regional disparities in access to such treatment continue to widen (Crime UNOoDa, 2023). Moreover, one consequence of the coronavirus 2019 pandemic has been a surge in the prevalence of DUDs (Dunlop et al., 2020). During the pandemic, the burden of DUDs in China has also worsened (Sun et al., 2020), highlighting the pervasive and global nature of this issue. Therefore, DUDs not only rank among the significant contributors to health and productivity decline among the global young and middle-aged population, but they also represent a clear and escalating threat that will continue to amplify the burden of disease and mortality risks in the coming decades.

The GBD database offers significant advantages for the systematic analysis and integration of global disease and health data. In the current global health landscape, obtaining the latest burden of disease information related to DUDs is crucial for national public health policies and healthcare services, aiding countries in formulating more effective policies for specific populations. However, existing research predominantly focuses on analyzing DUDs at the global or regional level, with insufficient studies addressing relevant data specific to China. Currently, only a study by Jianbo et al. has reported a declining trend in the risk of death and disease burden from DUDs in China, based on their analysis of the burden of substance abuse in 2019 using data from 204 countries and regions (Chen A. et al., 2022). Nonetheless, more in-depth research focusing specifically on China remains lacking. Therefore, in this study, we retrieved detailed data on the latest burden of DUDs from the 2021 GBD database to comprehensively investigate the magnitude and temporal trends of the burden of DUDs in China, overall and stratified by age and gender, from 1990 to 2021. Additionally, we further projected the trends of DUDs burden up to 2050 to identify high-risk populations, thereby providing a scientific basis for policymakers in their decision-making and intervention measures.

### 1.1 Theoretical framework

This study is grounded in the GBD conceptual framework, which operationalizes health loss through composite metrics of DALYs, Years of Life Lost (YLLs), and Years Lived with Disability (YLDs). The GBD paradigm, rooted in population health theory, asserts that quantifying YLLs and YLDs collectively captures the societal impact of diseases such as DUDs. By applying this model to China’s epidemiological data from 1990 to 2021, we align with the social determinants of health perspective, wherein temporal trends in burden reflect the interactions between policy interventions, healthcare access, and behavioral risk factors. Joinpoint and Age-Period-Cohort analyses extend this theoretical foundation by decomposing trends in disease burden into age-specific effects, period-driven policy influences, and generational cohort risks—a triangulation approach endorsed by Bayesian epidemiological models for forecasting. This framework inherently prioritizes YLLs as the primary indicator for fatal drug-related harm, consistent with the GBD’s emphasis on mortality-driven burden assessments for DUDs, while acknowledging prevalence rates as contextual markers of persistent public health challenges.

## 2 Materials and methods

### 2.1 Study data

This cross-sectional study used data from the 2021 GBD database (https://vizhub.healthdata.org/gbd-results/). GBD has systematically conducted scientific evaluations of data on incidence, prevalence, mortality, DALYs, YLDs, and YLLs for various diseases and injuries. These evaluations rely on published literature, publicly available resources, and contributed datasets, ensuring data reliability and accuracy (Vos et al., 2020; GBD 2017 Risk Factor Collaborators, 2018). Incidence denotes the number of new cases over a specified period, while prevalence reflects all existing cases at a single point in time. DALYs quantify overall disease burden as the number of years lost due to illness, disability, or premature death; specifically, DALYs = YLLs + YLDs. YLLs represent the number of years lost due to premature mortality, calculated by comparing age at death with a standard life expectancy. YLDs indicate the number of years lived in less-than-full health due to disease or injury, weighted by severity: YLDs = number of cases × average duration × disability weight (0–1) (Vos et al., 2020; Shao et al., 2023).

The specific methods for selecting data from the 2021 GBD database are as follows: the region specified as “China,” the cause of death as “drug use disorder,” gender categories include “both,” “female,” and “male,” a timeframe extending from 1990 to 2021, and age groups divided into 5-year intervals, ranging from 0 years to 95 years and older. In the GBD study (2021), DUDs were diagnosed according to the criteria outlined by the Diagnostic and Statistical Manual of Mental Disorders, Fourth Edition, Text Revision (DSM-IV-TR) or the International Classification of Diseases, 10th Revision (ICD-10). This diagnosis covers opioid, cocaine, cannabis, and amphetamine use disorders, along with other DUDs (GBD 2021 Diseases and Injuries Collaborators, 2024; Ong et al., 2024). Other SUDs included dependence on hallucinogens, inhalants or solvents, sedatives, tranquilizers, and various other drugs, substances, or intoxicants (GBD 2021 Diseases and Injuries Collaborators, 2024). This study did not require ethical approval or informed consent, as it utilized publicly accessible databases.

### 2.2 Statistical analysis

This study conducted a descriptive analysis of the trends in the disease burden of DUDs in China from 1990 to 2021. Specifically, it involved a comparative analysis of extracted data regarding DUD morbidity, mortality, and DALYs. The Joinpoint Regression Program was used to calculate the Average Annual Percentage Change (AAPC), and Age-Period-Cohort analyses were conducted to reflect the trends in the burden of DUDs. The Bayesian model for age-period-cohort was introduced to forecast the burden.

Trend analysis was conducted using a Joinpoint regression model, as described in prior literature (Helgadottir et al., 2024). The analysis computed the annual percentage change (APC), AAPC, and corresponding 95% confidence intervals (CIs). The Joinpoint Regression Program (version 5.1.0), developed by the Surveillance Research Program of the U.S. National Cancer Institute to detect trends and identify significant shifts in data trajectories over time. This method utilizes a permutation-based approach to establish joinpoints—critical junctures where trends change in direction or rate.

Age-Period-Cohort modeling was used to estimate the individual impacts of age, period, and birth cohort on the burden of DUDs. As comprehensively described in a previous study (Spittal et al., 2024), this approach enables a detailed exploration of the interplay among these three interrelated factors in influencing disease incidence and mortality rates. Simultaneously, a Bayesian Age-Period-Cohort (BAPC) model was developed to project the disease burden of DUDs up to 2050 (Chen J. et al., 2022; Fang et al., 2023; Knoll et al., 2020). The Bayesian inference used integrated nested Laplace approximation (INLA) with default priors, including Normal (0, 1/1000) for fixed-effect coefficients (including intercept) and Gamma (0.001, 0.001) for precision parameters (e.g., residual, random effects precision). This methodology is commonly used for forecasting disease incidence and mortality trends (Fan et al., 2023) and employs a log-linear Poisson model, assuming multiplicative effects among age, period, and cohort factors.

To address overdispersion, a negative binomial distribution for count data was implemented, along with a second-order random walk prior for smoothing age/period/cohort effects, as expressed in the formula: nij = log (λij) = μ + αi + βj + γk, where λij represents the number of cases, μ is the intercept, and αi, βj, and γk denote represent the effects of age, period, and cohort, respectively. The predicted population for the years 2022–2050 was obtained from the World Population Prospects 2022 (United Nations Department of Economic and Social Affairs PD, 2022) and adjusted for age stratification to align with the model’s requirements. Specifically, age groups were reorganized into 5-year intervals. This adjusted data was subsequently merged with historical population data to ensure a continuous time series from 1990 to 2050. The merged data was transformed into a wide format and used as weights within the model to adjust for age-standardized rates, thereby facilitating accurate prediction of incidence rates while accounting for changes in population structure over time.

All statistical analyses and data visualization were performed using R (version 4.3.2), Joinpoint (version 5.1.0), and the Age-Period-Cohort web tool (https://analysistools.cancer.gov/apc/). P-values <0.05 were considered statistically significant.

## 3 Results

### 3.1 Burden of DUDs in China in 2021 and temporal trend

The numbers for all ages and age-standardized rates of disease burden for overall and categorized by sex are presented in Table 1. In 2021, the age-standardized rates (per 100,000 people) of incidence, prevalence, mortality, DALYs, YLDs, and YLLs for DUDs were 173.24, 587.42, 0.69, 116.47, 83.90, and 32.57, respectively. Notably, YLDs accounted for 72% of total DALYs (83.90/116.47), indicating a disproportionate burden of disability in relation to YLLs within the population. The disease burden was substantially greater in males than females across all metrics, underscoring male-specific risk pathways.

**TABLE 1:** All-age cases and age-standardized prevalence, incidence, deaths, YLLs, YLDs, and DALYs rates in 2021 for drug use disorders in China.

Measure	All-age cases			Age-standardized rates per 100,000 people		
	Male	Female	Total	Male	Female	Total
Incidence	1289856 (1087513, 1513407)	1161458 (957931, 1396304)	2451314 (2046472, 2907371)	180.21 (151.7, 208.57)	165 (136.37, 195.85)	173.24 (145.45, 203.89)
Prevalence	4423772 (3796661, 5224514)	3256287 (2830653, 3816114)	7680059 (6602083, 9057281)	660.17 (553.41, 793.07)	505.84 (425.7, 607.18)	587.42 (492.04, 702.02)
Deaths	9106 (7063, 11554)	2377 (1816, 3071)	11483 (9278, 13907)	1.1 (0.86, 1.38)	0.29 (0.22, 0.37)	0.69 (0.56, 0.83)
DALYs	1021678 (795975, 1249554)	639530 (471657, 805253)	1661208 (1278814, 2030012)	139.44 (108.82, 171.93)	91.77 (66.46, 116.92)	116.47 (89.13, 143.59)
YLDs	621768 (432449, 814638)	536386 (371835, 695415)	1158154 (807684, 1501703)	89.08 (61.69, 118.43)	77.9 (52.99, 102.21)	83.9 (57.35, 110.53)
YLLs	399910 (309319, 503447)	103144 (78220, 134375)	503054 (406979, 605721)	50.36 (39.02, 63.09)	13.87 (10.47, 18.14)	32.57 (26.33, 38.92)

DALYs, disability-adjusted life-years; YLDs, years lived with disability; YLLs, years of life lost.

### 3.2 Burden and temporal trends of DUDs in China by age and sex

**FIGURE 1:**
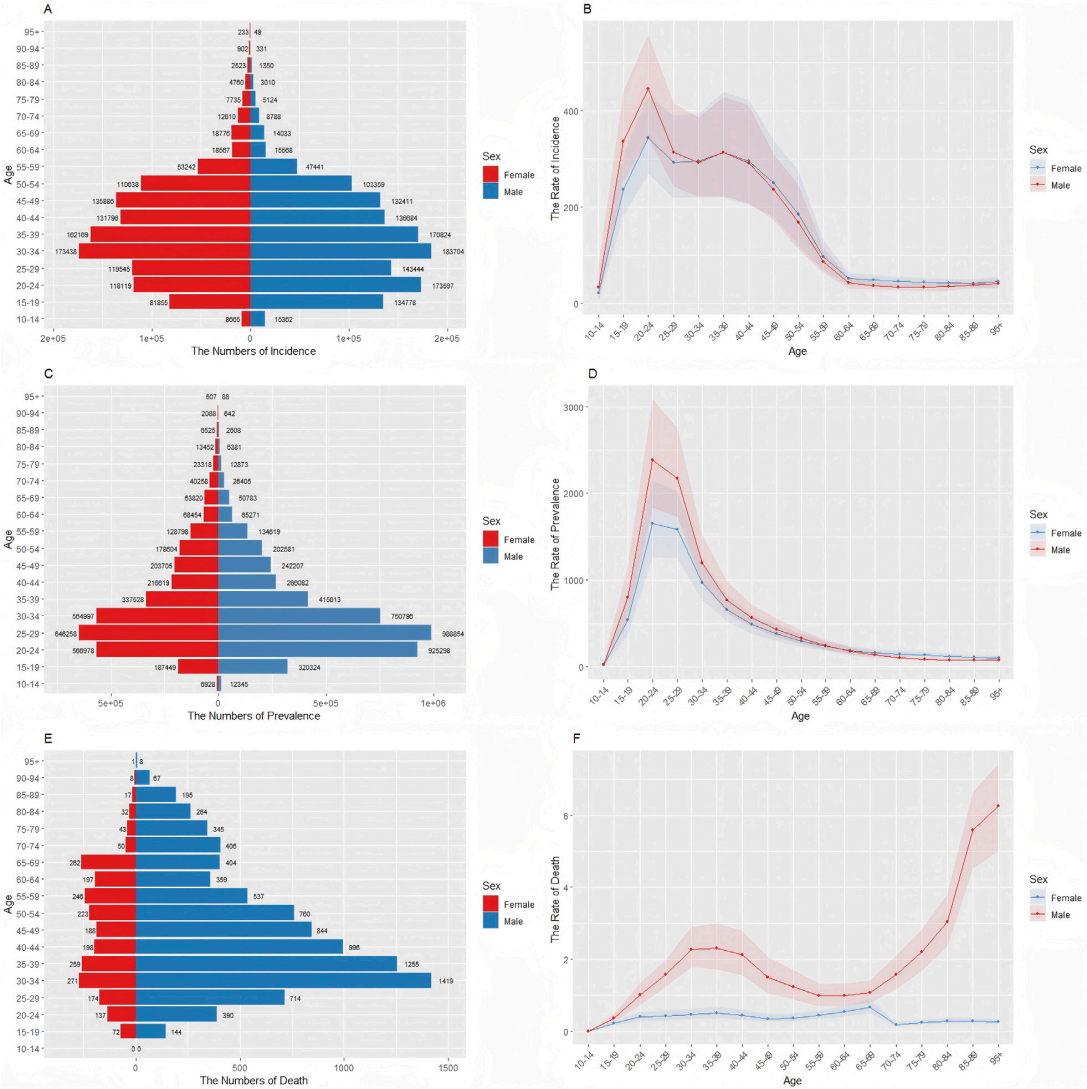
Age-specific numbers and age-standardized incidence, prevalence, and mortality rates of drug use disorders in China, 2021. **(A)** Age-specific incidence number. **(B)** Age-standardized incidence rate. **(C)** Age-specific prevalence number. **(D)** Age-standardized prevalence rate. **(E)** Age-specific mortality number. **(F)** Age-standardized mortality rate.

Figure 1 shows the numbers and age-standardized rates of incidence, prevalence, and mortality across different age groups in China for 2021. Both incidence and prevalence increased rapidly after the 10–14 age group peak at ages of 25–29 and 30–34 years. This aligns with typical onset ages for substance initiation and progression to disorder, reflecting critical windows for prevention. Peak rates occurred earlier (20–24 years) than peak case volumes, suggesting that young adults experience the highest *per capita* disease impact. Gender differences in mortality were pronounced: female deaths remained low and stable until age 69, while male deaths peaked at 30–34 years—reflecting riskier use patterns—before sharply rising after age 65, potentially indicating age-related comorbidities exacerbating drug-related mortality.

**FIGURE 2:**
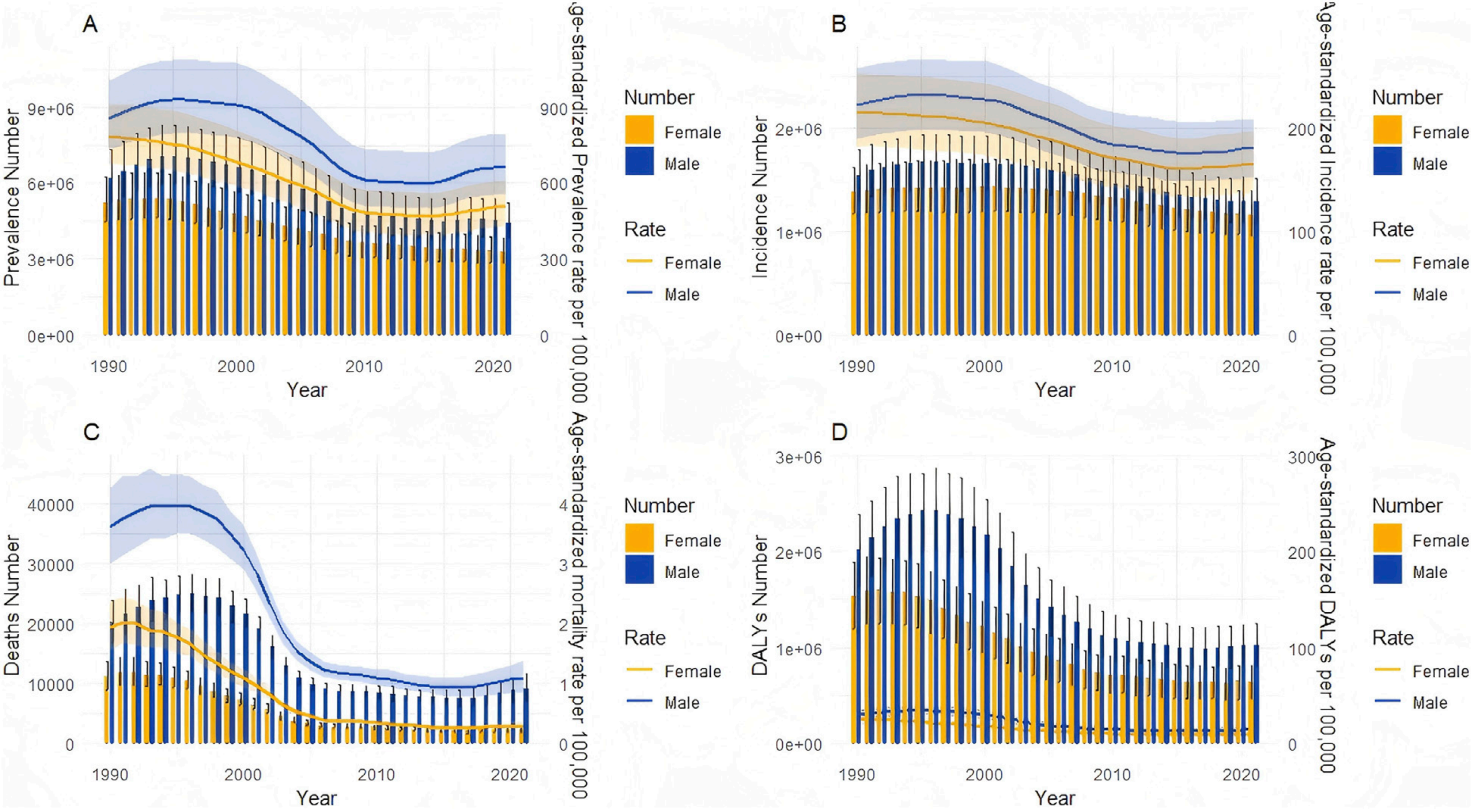
Trends in the all-age cases and age-standardized prevalence, incidence, mortality, and DALYs rates of drug use disorders by sex from 1990 to 2021. **(A)** Incidence number and rate. **(B)** Prevalence number and rate. **(C)** Mortality number and rate. **(D)** DALYs number and rate.

Figure 2 depicts the trends in prevalence, incidence, mortality, and DALYs from 1990 to 2021. A distinct epidemic wave emerged: the burden increased throughout the 1990s, peaked around 2000, and then declined until 2015. This pattern correlates with China’s intensified anti-drug campaigns and restrictions on opioid prescribing implemented post-2000. The subsequent slight increase observed after 2015 coincides with the emergence of synthetic opioids and stimulants, indicating new epidemiological challenges (NMPA, 2016).

### 3.3 Joinpoint regression analysis

According to the results from Joinpoint regression analysis, the Age-Standardized Incidence Rate (ASIR), Age-Standardized Prevalence Rate (ASPR), and Age-Standardized Mortality Rate (ASMR) for DUDs in China exhibited a general decrease from 1990 to 2021, with the AAPCs (95% CI) of −0.76 (−0.83, −0.69), −1.05 (−1.25, −0.84), and −4.41 (−4.75, −4.08), respectively (Table 2). We found the ASIR trend exhibited slight increases during 1990–1995 (APC = 0.35) and 2015–2021 (APC = 0.51), and while it decreased substantially from 1995 to 2015, particularly during 2001–2006 (APC = −2.05) and 2006–2009 (APC = −2.51). Similarly, the ASPR trend showed increases during 1990–1995 (APC = 0.68) and 2014–2021 (APC = 1.62), with a decrease observed from 1995 to 2014, especially in the periods 2001–2006 (APC = −3.46) and 2006–2009 (APC = −5.65). The ASMR trend exhibited slight increases in 1990–1995 (APC = −3.46) and a significant increase during 2016–2021 (APC = −3.46) while demonstrating a notable decrease from 1995 to 2016, particularly in the interval from 2000 to 2005 (APC = −3.46). It is noteworthy that the rebound of ASIR and ASPR after 2015 (with APC values of 0.51 and 1.62) was closely associated with trends in synthetic drug epidemics in China, whereas the continued decline of ASMR (with an AAPC of −4.41) reflected the diminishing dominance of opioid-related fatalities.

**TABLE 2:** Trends in age-standardized incidence, prevalence, mortality rates (per 100,000 persons) among both sexes, males, and females in China, 1990–2021 for drug use disorders in China.

Gender	Age-standardized incidence rates			Age-standardized prevalence rates			Age-standardized mortality rates		
	Period	APC (95% CI)	AAPC (95% CI)	Period	APC (95% CI)	AAPC (95% CI)	Period	APC (95% CI)	AAPC (95% CI)
Both	1990–1995	0.35 (0.23, 0.47)	−0.76 (−0.83, −0.69)	1990–1995	0.68 (0.35, 1.02)	−1.05 (−1.25, −0.84)	1990–1995	0.78 (−0.19, 1.75)	−4.41 (−4.75, −4.08)
	1995–2001	−0.57 (−0.69, −0.45)		1995–2001	−1.22 (−1.55, −0.88)		1995–2000	−5.41 (−6.39, −4.42)	
	2001–2006	−2.05 (−2.23, −1.88)		2001–2006	−3.46 (−3.95, −2.96)		2000–2005	−17.53 (−18.38, −16.67)	
	2006–2009	−2.51 (−3.07, −1.94)		2006–2009	−5.65 (−7.22, −4.05)		2005–2016	−3.54 (−3.83, −3.26)	
	2009–2015	−1.17 (−1.30, −1.04)		2009–2014	−0.98 (−1.52, −0.43)		2016–2021	4.09 (2.66, 5.54)	
	2015–2021	0.51 (0.41, 0.61)		2014–2021	1.62 (1.39, 1.86)				
Female	1990–1997	−0.32 (−0.39, −0.25)	−0.85 (−0.92, −0.78)	1990–1996	−0.73 (−0.89, −0.58)	−1.37 (−1.50, −1.25)	1990–1995	−2.02 (−3.63, −0.39)	−6.10 (−6.65, −5.56)
	1997–2001	−0.82 (−1.08, −0.56)		1996–2001	−2.21 (−2.50, −1.91)		1995–2001	−9.88 (−11.01, −8.73)	
	2001–2006	−1.86 (−2.03, −1.70)		2001–2006	−3.14 (−3.44, −2.84)		2001–2005	−18.81 (−20.88, −16.68)	
	2006–2009	−2.18 (−2.71, −1.65)		2006–2009	−5.07 (−6.04, −4.10)		2005–2016	−4.67 (−5.09, −4.24)	
	2009–2015	−1.34 (−1.46, −1.22)		2009–2015	−0.81 (−1.05, −0.57)		2016–2021	2.70 (0.51, 4.94)	
	2015–2021	0.52 (0.43, 0.61)		2015–2021	1.55 (1.36, 1.73)				
Male	1990–1995	0.86 (0.73, 1.00)	−0.69 (−0.76, −0.61)	1990–1994	2.16 (1.53, 2.78)	−0.79 (−1.05, −0.53)	1990–1996	1.46 (0.80, 2.14)	−3.83 (−4.14, −3.52)
	1995–2001	−0.46 (−0.59, −0.33)		1994–2001	−0.39 (−0.71, −0.06)		1996–2000	−4.68 (−6.10, −3.25)	
	2001–2006	−2.17 (−2.36, −1.98)		2001–2006	−3.68 (−4.29, −3.06)		2000–2005	−17.27 (−17.98, −16.55)	
	2006–2009	−2.90 (−3.51, −2.29)		2006–2009	−6.14 (−8.13, −4.12)		2005–2016	−3.26 (−3.50, −3.01)	
	2009–2016	−0.91 (−1.02, −0.80)		2009–2014	−0.93 (−1.63, −0.22)		2016–2021	4.21 (2.92, 5.52)	
	2016–2021	0.66 (0.52, 0.81)		2014–2021	1.74 (1.44, 2.04)				

DALYs, disability-adjusted life-years; YLDs, years lived with disability; YLLs, years of life lost.

**FIGURE 3:**
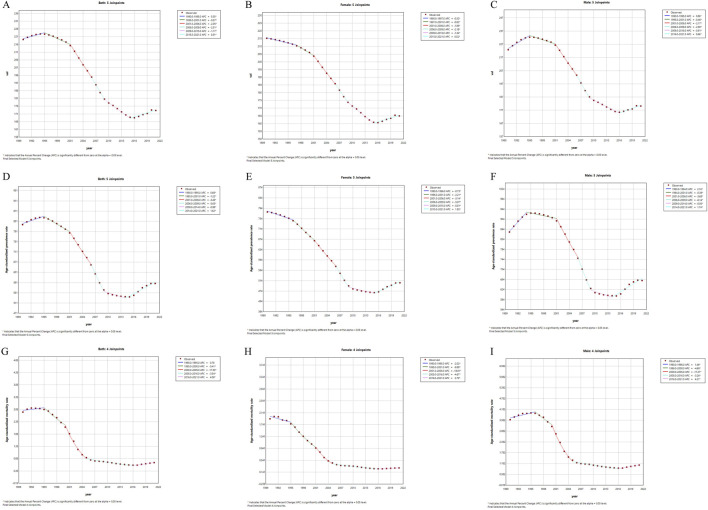
The sex-specific age-standardized incidence, prevalence, mortality rates (per 100000 persons) for drug use disorders in China from 1990 to 2021. **(A)** age-standardized incidence rates in female and male; **(B)** age-standardized incidence rates in female; **(C)** age-standardized incidence rates in male; **(D)** age-standardized prevalence rates in female and male; **(E)** age-standardized prevalence rates in female; **(F)** age-standardized prevalence rates in male; **(G)** age-standardized mortality rates in female and male; **(H)** age-standardized mortality rates in female; **(I)** age-standardized mortality rates in male.

Figure 3 illustrates the trends in age-standardized rates of the burden of substance use disorders by sex in China from 1990 to 2021. The findings indicate an overall trend of “increase-peak-decrease-rebound” in the burden of DUDs in the country. Regarding incidence and prevalence, the ASIR and ASPR for males experienced a brief increase from 1990 to 1995, followed by a continuous decline, with a rebound observed after 2016 (Figures 3C,F). Conversely, the ASIR and ASPR for females have exhibited a gradual decline since 1990, with a slight increase noted after 2016 (Figures 3B,E). Notably, the decline in male ASPR from 1994 to 2001 was minimal (APC = −0.39%), whereas the decline for females during the same period was more pronounced (APC = −2.21%). Regarding mortality rates, the ASMR for males showed a slight increase from 1990 to 1995 before entering a phase of continuous decline (Figure 3I), while the ASMR for females decreased consistently over time (Figure 3H).

### 3.4 Analysis of age-period-cohort models for the incidence and mortality rates of DUDs in China

**FIGURE 4:**
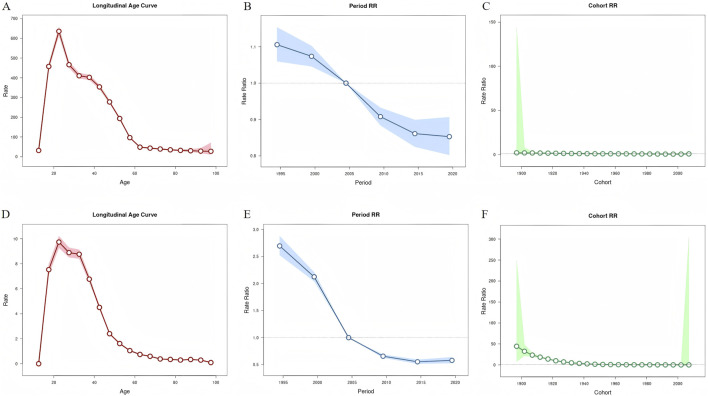
Age, period and cohort effects on drug use disorders incidence and mortality rate in China during 1990–2021. **(A–C)** The age, period and cohort effects for incidence rate; **(D–F)** the age, period and cohort effects for mortality rate.

Figure 4 presents the effects of age, period, and birth cohort on DUDs. The effects of age on incidence and mortality related to DUDs gradually increased from the 10–14 age group, peaking in the 20–24 age group. After this peak, rates sharply declined until the 45–50 age group, remaining relatively stable thereafter. The effect of period on incidence and mortality rates decreased at a relatively constant rate over time. Compared with the period effect in 2002–2006, the highest relative risks (RRs) (95% CI) for incidence and mortality during the period 2002–2006 were 1.11 (1.06, 1.15) and 2.70 (2.53, 2.88) in 1992–1996, respectively. Conversely, the lowest RRs (95% CI) of incidence and mortality were 0.85 (0.80, 0.91) and 0.58 (0.53, 0.64) during 2017–2021, respectively.

The cohort effects on incidence and mortality showed a continuous downward trend in risk from earlier to later birth cohorts. Compared to the cohort effect observed in the 1952 birth cohort, the highest RRs (95% CI) of incidence and mortality were 2.11 (0.03, 144.44) and 44.33 (7.86, 249.96) in the 1987 birth cohort, respectively. In contrast, the lowest RRs (95% CI) of incidence and mortality were 0.83 (0.70, 0.98) and 0.06 (0.01, 304.36) in the 2007 birth cohorts, respectively.

### 3.5 Prediction of the DUDs-related burden in China over the next 30 years based on the BAPC model

The sex-specific epidemiological trends of DUD in China were projected from 2022 to 2050 using the BAPC prediction model. Generally, both the ASIR and ASPR were expected to continue rising for both females and males, while the ASMR was not expected to change significantly.

Specifically, the ASIR for females was expected to increase from 166.58 per 100,000 in 2022 to 407.85 per 100,000 in 2050, and for males, from 185.00 per 100,000 in 2022 to 642.50 per 100,000 in 2050. Similarly, the ASPR was projected to increase from 526.54 per 100,000 in 2022 to 1,601.32 per 100,000 in 2050 for females, and from 691.35 per 100,000 in 2022 to 2,264.83 per 100,000 in 2050 for males. However, the ASMR was projected to remain relatively stable, ranging from 0.24 to 0.32 per 100,000 for females and from 1.6 to 2.4 per 100,000 for males between 2022 and 2050.

**FIGURE 5:**
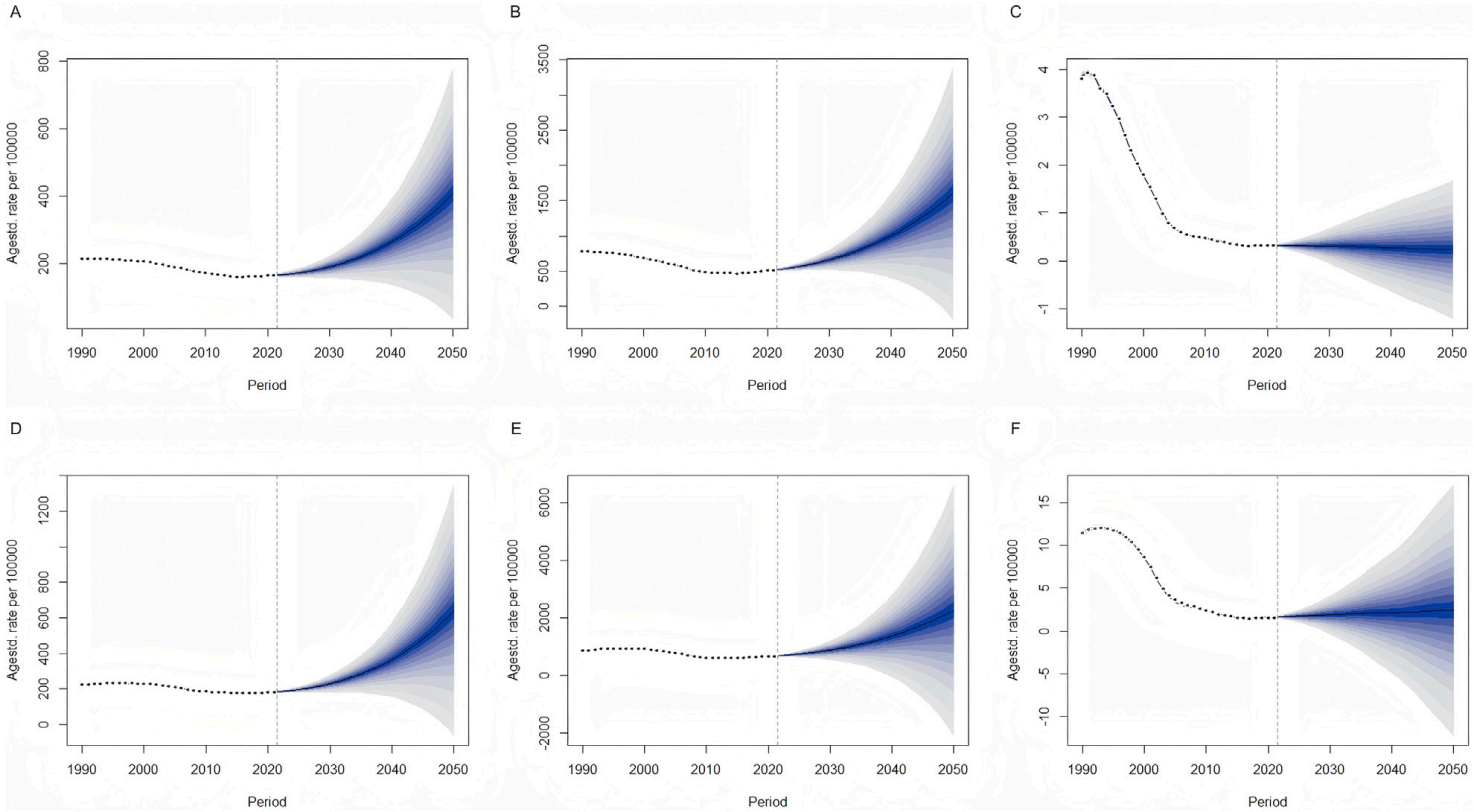
Trends of sex-specific age-standardized incidence, prevalence, mortality rates (per 100000 persons) for drug use disorders from 2021 to 2050 predicted by Bayesian age-period-cohort (BAPC) models. **(A)** age-standardized incidence rates in female; **(B)** age-standardized prevalence rates in female; **(C)** age-standardized mortality rates in female; **(D)** age-standardized incidence rates in male; **(E)** age-standardized prevalence rates in male; **(F)** age-standardized mortality rates in male.

Prediction intervals were calculated to provide a quantitative assessment of the uncertainty in our model’s forecasts. As depicted in Figure 5, these intervals are illustrated by shaded areas surrounding the forecast lines, indicating the 95% prediction intervals and the credible range of predicted values. The widening of these intervals as the projection period extends into the future underscores the increasing uncertainty inherent in long-term forecasts.

## 4 Discussion

DUDs are classified as chronic and recurrent neurological conditions that disrupt normal brain function by modifying reward pathways and synaptic plasticity (Cheron et al., 2021). Excessive substance use can lead to significant disability or even death. This disorder profoundly affects both the physical and mental wellbeing of individuals, imposing a considerable economic strain on families and society at large. This study used data from the 2021 GBD database to systematically analyze the characteristics of the disease burden associated with DUDs in China from 1990 to 2021, as well as its temporal evolution and projections extending to 2050. The results indicate a notable decrease in both the mortality rate and DALYs associated with DUDs in China from 1990 to 2021. Nevertheless, the absolute incidence and prevalence of DUDs continue to increase, with significant variations observed between genders. Notably, forecasts derived from the BAPC model indicate that the incidence and prevalence of DUDs are expected to increase substantially until 2050, while the mortality rate is projected to remain relatively stable. These findings offer critical insights for policymakers to formulate targeted prevention and intervention strategies.

The findings of this study reveal that the ASIR, ASPR, ASMR, and DALYs associated with DUDs in China exhibited an overall trend of initially increasing, then decreasing from 1990 to 2021. In the early 1990s, China faced a complex social environment characterized by a high availability and abuse of drugs (Wang et al., 2018), coupled with inadequate medical resources, which led to a higher risk of morbidity and mortality associated with DUDs. This study demonstrates that incidence, prevalence, mortality rates, and DALYs increased from 1990 to 1995. This finding aligns with the declining trends in mortality rates and DALYs burden for DUDs reported in the 2021 GBD report (Zhang et al., 2024). Entering the late 2000s, standardized incidence rates, standardized mortality rates, and standardized DALYs for DUDs exhibited a significant downward trend. This decline can be attributed to the long-term efforts of the Chinese government and associated agencies in combating DUDs, which included comprehensive measures such as policy implementation interventions, promotion of mental health education, improvement of medical resource allocation, and control of illegal drug use (Li et al., 2010; Lu et al., 2008; Luo et al., 2019; Yang and Giummarra, 2021; Liu et al., 2017). These efforts greatly reduced the accessibility of drugs, thus lowering the risks of disability or death. Notably, there has been a resurgence in the ASIR and ASPR for DUDs in China following 2015. Both male and female ASIR and ASPR exhibited a slight upward trend post-2015, potentially linked to rising rates of opioid abuse (Zhong et al., 2019), increases in the use of synthetic drugs (including methamphetamine, 3,4-methylenedioxymethamphetamine, and ketamine) (Bao et al., 2015; Palamar, 2020; Li et al., 2021), and the rapid proliferation of new psychoactive substances (such as cathinones and synthetic cannabinoids) (Hou et al., 2024). The high prevalence of depression and anxiety, coupled with increasing social pressures, serve as critical factors (Ruan et al., 2024). Mental health challenges can act as both triggers for DUDs and exacerbating influences during drug withdrawal. In 2015, policy adjustments were made, including the expansion of mandatory urine screening and the inclusion of new psychoactive substances in the detection list (MoP, 2015), thereby enhancing the capacity for case detection. Nevertheless, drug control data showed a more than 300% increase in the seizure volume of synthetic drugs during the same period (NMPA, 2016), confirming a substantive epidemiological basis for the observed resurgence in incidence rates. The sensitivity of monitoring is compounded by the actual prevalence, creating a synergistic enhancement effect.

The findings of this study indicate that, in China, the rates of DUD-related ASIR, ASPR, ASMR, YLLs, YLDs, and DALYs are higher in males than in females. The burden of DUD-related diseases also appears to be more severe among males across most age groups. These data suggest that the burden of DUD-related diseases in China aligns with global patterns (Sugimoto et al., 2025), with expected higher indicators among Chinese males than females. Factors contributing to this disparity may include biological sex differences (such as variations in brain structure and function, endocrine function, and metabolic function) (Steingrímsson et al., 2012; Marinelli et al., 2023), differing social roles (Becker et al., 2016), and a greater likelihood of males consuming higher doses of psychoactive substances (Moryl et al., 2018). Additionally, environmental factors and disparities in healthcare responses may exacerbate these gender-related differences in burden (Marsh et al., 2010). However, further research should be conducted in China to measure the burden of DUDs across various sub-populations. Such studies could inform the design and implementation of specific treatment and prevention programs aimed at reducing the disease burden associated with DUDs.

This study provides an in-depth analysis of the burden of substance abuse disorders across different age groups in China. The results indicate that the standardized incidence and prevalence rates of substance abuse rose sharply after the 10–14 age group, peaking within the 20–24 age group, followed by a declining trend with increasing age. This pattern is consistent with the epidemiological characteristics of high prevalence of DUDs observed globally (Zhang et al., 2024). Adolescent substance abuse is a significant public health issue worldwide. In numerous countries, the prevalence of DUDs and DALYs among adolescents is notably high (Lin et al., 2024). In China, the use of psychoactive substances, including tobacco, sedatives, analgesics, and illicit drugs, is prevalent among individuals aged 10–24 (RJWBA et al., 2017). Additionally, the non-medical use of prescription drugs (such as analgesics and stimulants) is common among college students (Tam et al., 2018), highlighting a severe concern surrounding substance abuse among adolescents. Contributing factors include mental health challenges, family dynamics, socioeconomic pressures, lack of educational opportunities, and poor employment prospects (Nath et al., 2022; Torres et al., 2020; He et al., 2008; Casal et al., 2025; Kuhn, 2015; Khoddam et al., 2018). The earlier the onset of psychoactive substance use, the greater the lifetime health risks associated with substance abuse (Silveri et al., 2016; Conrod and Nikolaou, 2016). Consequently, it is recommended that preventive measures and interventions be implemented early among youths to mitigate the health burden attributable to substance abuse.

This study also found that the standardized mortality rate for DUDs among women remained consistently low across all age groups, while the standardized mortality rate for males exhibited a bimodal distribution, with peaks occurring within the 30–34 years and over 70 years age groups. Several factors contribute to the elevated mortality rate from DUDs among middle-aged men. On one hand, this demographic is particularly susceptible to pressures from socioeconomic or occupational stress, family responsibilities, and changes in social or familial dynamics, which may serve as potential triggers for drug use (Jones et al., 2023). Conversely, individuals in this age group often encounter barriers to accessing treatment for DUDs due to social stigma, fear of occupational repercussions, and family obligations, leading to delays in obtaining timely treatment or proper management. The cumulative damage resulting from prolonged drug exposure, coupled with inadequate treatment or management, may contribute to the elevated mortality rates associated with DUDs in this population (Guo et al., 2022). The unusually high mortality rate from DUDs among individuals over 70 years of age may relate to comorbid physical conditions in elderly men (Su et al., 2023), necessitating the long-term use of prescription and over-the-counter medications that can lead to both medical and non-medical DUDs, creating a vicious cycle that ultimately results in increased mortality from DUDs in older men (Koechl et al., 2012). Additionally, social isolation, loss of roles, anxiety, depression, and other mental health issues may drive individuals to seek solace in drugs (Hyde et al., 2015; Leinonen et al., 2013; Lam and Vuolo, 2022). Thus, there is an urgent need to enhance attention and interventions regarding DUDs among the elderly to mitigate the associated mortality rates.

The BAPC model predict a steady increase in the ASIR and ASPR for DUDs within the Chinese population from 2022 to 2050. This upward trend is evident among both genders, with a higher annual growth rate of 4.54% for males compared to 3.25% for females. Such projections suggest that DUDs may emerge as a significant public health concern in China over the next 28 years, highlighting the need for targeted efforts to address DUDs in men. The repercussions of DUDs extend beyond individuals, adversely affecting quality of life, work performance, familial relationships, and social interactions, while also imposing considerable health and economic burdens (Rostam-Abadi et al., 2023). Notably, the projected divergence between rising incidence/prevalence and stable mortality rates underscores a pivotal epidemiological shift towards low-lethality synthetic drugs (e.g., cathinones, synthetic cannabinoids), necessitating strategic policy recalibration. Interventions must prioritize youth-focused digital prevention campaigns tailored to the neurodevelopmental vulnerabilities between ages 15 and 24 years, while rehabilitation programs urgently require retooling from opioid-centric protocols to cognitive-behavioral therapies aimed at addressing stimulant-induced psychosis. Simultaneously, integrating mandatory screening in high-risk male-dominated industries such as construction and logistics could play a critical role in intercepting early-stage addiction, leveraging workplace infrastructure for scalable prevention.

While this study presents the trends in the burden of DUDs in China from 1990 to 2021 and forecasts potential changes in burden among male and female populations up to 2050, it is important to acknowledge certain limitations. First, case ascertainment in the GBD 2021 framework relies on diagnostic criteria codified in the DSM-IV-TR and ICD-10 classification systems. The recent nosological shift to DSM-5 introduces challenges related to diagnostic reclassification that could significantly alter prevalence estimates. Second, the model-generated epidemiological parameters derived from GBD’s hierarchical estimation framework—which incorporates system dynamics models and Bayesian meta-regression tools—remain contingent upon the quality of input data. Potential gaps in data completeness and population representativeness may introduce estimation bias, particularly given the inherent concealment characteristics associated with substance use behaviors. Third, formal out-of-sample validation and sensitivity analyses for BAPC projections were not performed; although the Bayesian framework of the model inherently quantifies uncertainty through posterior credible intervals, retrospective validation remains constrained by data limitations in the full-cohort historical series of the GBD. Future research will prioritize temporal hold-out validation of the BAPC model to further assess its predictive robustness. Fourth, while GBD 2021 provides standardized global estimates, its reliance on aggregated secondary data often overlooks marginalized groups—such as incarcerated or rural drug users—relevant to China’s drug landscape. Finally, although this study evaluates trends in the burden of DUDs over nearly 30 years based on the 2021 GBD data and predicts future burdens, the information lag in this database—currently limited to data spanning only from 1990 to 2021—suggests that the prediction results may not be entirely accurate. Nonetheless, the findings of this study possess significant implications for public health efforts aimed at controlling the burden of DUDs in China.

## 5 Conclusion

Despite the overall decline in the disease burden of DUDs in China from 1990 to 2021, these disorders persist as a critical public health challenge, particularly given the country’s extensive population base and the disproportionate burden observed among males. While the historical downward trend reflects the efficacy of previous drug control efforts, our projection models indicate a clear shift: both the incidence and prevalence of DUDs in China are anticipated to continue rising through 2050. These findings underscore the urgent necessity for targeted and proactive intervention strategies that are tailored to counter this emerging upward trend and to mitigate the growing disease burden associated with DUDs.

## Data Availability

The original contributions presented in the study are included in the article/supplementary material, further inquiries can be directed to the corresponding authors.

## Abbreviations

AAPC, Average Annual Percentage Change; APC, Annual Percentage Change; ASIR, Age-Standardized Incidence Rate; ASMR, Age-Standardized Mortality Rate; ASPR, Age-Standardized Prevalence Rate; CI, Confidence interval; DALY, Disability-Adjusted Life Years; DUD, Drug use disorders; GBD, Global Burden of Disease; INLA, Integrated nested Laplace approximation; YLD, Years Lived with Disability; YLL, Years of Life Lost.
